# Clinical Observations on the Early Stages of Trench Nephritis

**Published:** 1919

**Authors:** 


***Clinical Observations on the Early Stages of Trench Nephritis.*** By Major G. S. Strathy, C. A. M. C. *Bulletin of the Canadian Army Medical Corps*, September, 1918.

The author gives a short history of cases of epidemic trench nephritis observed since the outbreak of the war, and records the various suggestions which have been made concerning the causative and predisposing agents of the disease. Microbian infection is most probably the cause of trench nephritis, and although no conclusion has yet been reached, as far as the predisposing factors are concerned, the following facts may be noted: The epidemic is more prevalent on the West-European front, and the percentage of cases higher in the British than in the French armies, although the disease is non-existent in troops before landing in France or other theatres of war. Out of 300 cases admitted to a given C. C. S., only 2 were officers. Several Australians developed the first symptoms on board ship coming from Egypt.

Cases of trench nephritis may be grouped as follows:

(1) Mild cases with albuminuria and a few subjective symptoms. It is with difficulty that these are differentiated from albuminuria due to other causes.

(2) Hematuria with symtoms pointing to affection of the lower part of the urinary tract.

(3) Cases of nephritis with edema (all severe cases).

A number of cases illustrating each of the above three categories are given in detail.

*Treatment.* The general treatment is rest in bed and a simple diet of milk and milk puddings. Hot-air baths are recommended in cases of marked edema; the bowels must be kept free. In cases with severe dyspnea, oxygen inhalations are valuable. For convulsions, spinal punctures have given good results.

*Prognosis.* The mortality is low, but no definite opinion can yet be formed as to the seriousness of the disease, for a certain length of time must elapse before it is possible to judge whether epidemic trench nephritis has a tendency to become chronic, as is the case in acute nephritis of general practice.

